# Case Report: Account of chickenpox progression over 10 days

**DOI:** 10.12688/f1000research.124959.1

**Published:** 2022-09-06

**Authors:** Gudisa Bereda

**Affiliations:** 1 Pharmacy, Negelle health science college, Negelle, Oromia regional state, 1000, Ethiopia

**Keywords:** Case reports, Chickenpox infections, Healthcare workers, Acyclovir, progression, Varicella-zoster virus

## Abstract

Chickenpox is an extremely contagious disease; caused by the varicella-zoster virus primary infection. A 27-year-old adult Black African male health care worker presented with severe headache, intermittent weakness and inability to walk, intermittent nausea, fever, nocturnal polydipsia, shortness of breath, itching, pruritus (intensely pruritic erythematous macules), lesions with pus on the skin, sleep disturbances, and nightmares for two days. The most commonly occurring symptom of chickenpox is a vesicular rash that appears on the scalp, back and front of the neck, face and scapulae, and then disseminates distally to the limbs. In this case report, the patient face, neck and scapulae were the most infected areas of the body and the rest of the body except the legs, hands, genital areas and buttocks, were also highly infected. Acyclovir 800 mg orally, five times a day was given for ten days to cure chickenpox infection because acyclovir inhibits the replication of the varicella zoster virus, and has the ability to eradicate varicella zoster virus and relieve the symptoms more readily.

## Introduction

Chickenpox can be defined as an extremely contagious disease; which happens as a result of varicella-zoster virus primary infection. ^1^^,^^2^ The chickenpox infection disease course can rarely be more severe than anticipated and spread to involve different organs and causes severe complications. ^3^ In healthy individuals chickenpox infection can be often a mild, self-limiting illness, described by fever, malaise, and a generalized itchy, and vesicular rash. ^4^ When comparing children’s clinical manifestations of chickenpox with adults’; in adults they are more severe and more frequently correlated with complications. ^5^^–^^7^ The rash is most frequently distributed over the trunk, scalp and face. ^8^ Chickenpox is spread by direct person-to-person contact of open lesions or airborne droplets, and tends to elevate in severity with each subsequent case within a household. ^9^ This case report demonstrates the clinical manifestations, sites of severe infections; and counts the numbers of rashes that occurred in successive days until peeled off in an adult.

## Case report

**Figure 1.**
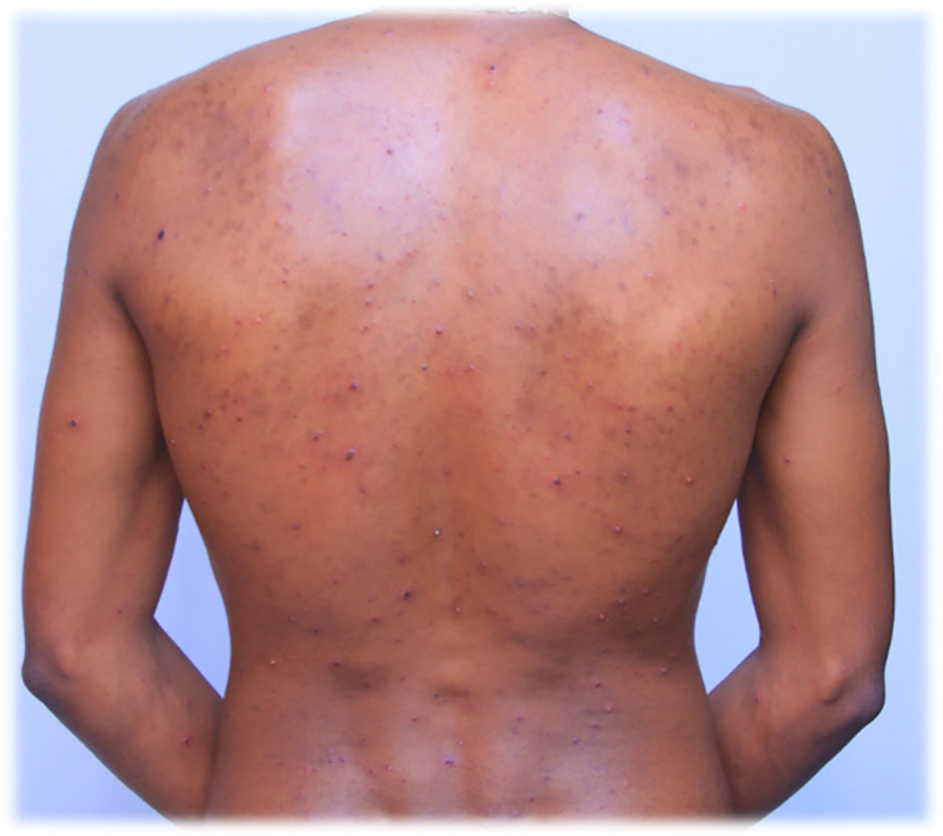
Red rash with ruptured lesions on the back, especially at the scapulae and lower back of the patient. This picture was captured at day three of the infection.

**Figure 2.**
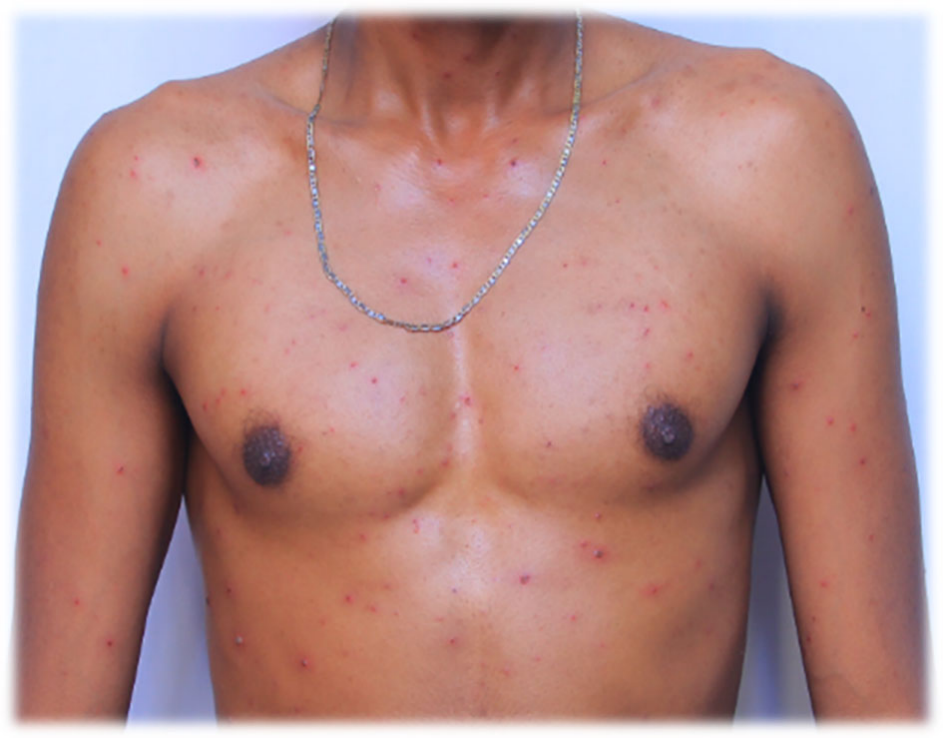
Red rash with ruptured lesions on the anterior body, especially on and around the chest of the patient. This picture was captured at day three of the infection.

A 27-year-old adult Black African male health care worker was admitted on July 28/2022. The admitted patient had no past medical and medication history and also no family medical and medication history. On admission, the patient presented with severe headache, intermittent weakness and inability to walk, intermittent nausea, fever, nocturnal polydipsia, shortness of breath, itching, pruritus (intensely pruritic erythematous macules), lesions with pus on the skin, sleep disturbances, and nightmares for two days. The patient was diagnosed based on the prodrome symptoms and the pattern of skin eruptions because of the inaccessibility of an immunofluorescence assay. The erythematous pruritic macules converted into clear fluid-filled vesicles on the face after 12 hours and on other infected areas such as the trunk, scalp, scapulae, back and lower extremities in 24 hours. The admitted patient was taking a shower in their work-place at 5:00pm with cold water and using soap and cloth in the shower room on July 25/2022; and the rare clinical manifestations such as fever and tiny lesions appeared on the face the morning after the shower. By the evening, lesions had appeared on several sites on the body, especially around the scapulae, neck and chest, and the number on the face had increased. On July 29/2022 the lesions or maculae on the face transformed to fluid filled vesicles, had increased in numbers to at least 165, and become enlarged in size. They had spread further on the body, especially to the scapulae and around the deltoid muscles, on the back and front of the neck, on the chest, and lower to the waist. The patient had developed a severe headache and experienced itching over the entire body. The red rashes were especially prominent around the back and front of the neck, with approximately 112 fluid filled vesicles. There was also one fluid filled vesicle around the genital area and three fluid filled vesicles on the buttock. The red rashes also rarely occurred in the outer ear, with at least seven fluid filled vesicles in and around the left outer ear and only one fluid filled vesicle in the right outer ear. Red rashes had developed on the scalp with at least 27 fluid filled vesicles. The red rash on the face appeared to be more severe than other sites because it was extremely enlarged compared to the other infected areas. The redness seen in Figures 1 and 2 also appeared on the face.

On July 30/2022 the fluid filled vesicles on the face dried and converted to hard rash (crusts), but on the upper and lower extremities the fluid filled vesicles stayed the same on all the body with intermittent severe headache and itching. The red rashes on the back of the patient increased in number to at least 215, and on the anterior of the body, especially on and around the chest, increased to around 85 fluid filled vesicles. On August 01/2022 the redness on the face had almost blackened and disappeared or changed to crusted or hard rashes and the redness elsewhere on the body had enlarged in size, and increased in numbers from July 30/2022 and started to appear on the legs and hands in small amounts. On August 02/2022 the red rash on the face completely peeled off and the previous vesicles on the face changed to a black color rather than the usual skin tone. The redness elsewhere on the body, except the face and neck somewhat decreased in size, and number, but it continued to spread on the legs and hands. On August 03/2022 the rash on the face totally disappeared and where the vesicles had previously appeared, the skin color blackened. The rash on the torso changed from a red colored rash to a black or crusted rash and started to decrease in size. On the hands and legs, the rash increased in size and multiplied in the number of vesicles to approximately 21 red rashes and 12 red rashes respectively.

On August 04/2022 the rash on the face had totally disappeared and the site of infection had turned to a black color, and on the rest of the body the rash sites had blackened except for the hands and legs. On August 05/2022 the rash on the body totally dried and converted the infected areas to the blackened color, but on the hands and legs the rash had only partially changed to a black color. On August 06/2022 the rashes on the hands and legs had become crusted with a black color at the infected sites. Finally on August 07/2022 morning, the crusted rashes on the hands, legs, genital area, and buttocks peeled off and made the infected area blackened. The patient was admitted to hospital for ten days and discharged back to his home on August 07/2022 at noon. The patient was treated by three medications; starting from the date of admission. I) Acetaminophen 500 mg orally, three times a day for five days was given to alleviate the fever or sores induced by chickenpox infection. II) Cetirizine hydrochloride 10 mg taken orally, was given two times a day for five days to minimize itching, and inhibiting the patient from scratching the rash and blisters, especially at night time. III) Acyclovir 800 mg orally, five times a day for ten days was given to cure the chickenpox infection because acyclovir inhibits the duplication of the varicella zoster virus, and has the ability to eradicate varicella zoster virus and relieve symptoms more readily. At discharge, zinc calamine lotion of 8% calamine and 8% zinc, was given to be applied two times a day for five days to relieve itchiness and also prevent further skin infections.

### Patient perspective

The patient’s symptoms were improved from the infection, and he was discharged back to his home with the prescribed medications.

## Discussion

Chickenpox is frequently experienced by children, with a peak incidence in those aged less than 10 years, but it is able to infect any age group. ^10^ The most commonly occurring symptom of chickenpox is a vesicular rash that appears on the scalp, face and trunk, and then disseminates distally to the limbs. ^11^^,^^12^ In this case report, the face, neck and scapulae were the most infected areas of the body, and the rest, except the legs, hands, genital areas and buttocks, were also highly infected. The legs, hands, genital areas and buttocks did not exhibit as much infection, especially the genital area where only one red rash appeared. There are three phases of clinical manifestations of chickenpox: I) the short prodrome illness, which appears 1-2 days after infection and comprises mild or moderate fever. II) The exanthematous phase, which comprises a rash; that appears from the first day. The first rash transformed into clear fluid-filled vesicles and later converted to a hard rash that became crusted as discussed above. At the exanthematous phase the rash more commonly appears on the scapulae, chest, and face and above and around the lower back. III) The final phase is the convalescent phase or remedial phase; at this phase the crusted usually resolves within seven days. ^13^ The main goal in the management of chickenpox infection is to alleviate the symptoms such as skin infections, fever, and itching and to make the individual comfortable. ^14^^,^^15^ Calamine lotion has skin-soothing properties, and can be used to relieve itching. ^16^ Acetaminophen is the preferred painkiller for the management of chickenpox correlated with fever because of its very rare risk of non-steroidal anti-inflammatory drugs induced skin blisters and rashes. ^17^^,^^18^ Cetirizine can alleviate itching and inhibit excoriation from happening. ^19^ For the treatment of chickenpox infection acyclovir was initiated at a dose of 800 mg orally, five times a day for ten days. Acyclovir works by preventing the replication of varicella-zoster virus. Acyclovir triphosphate is a competitive inhibitor of viral deoxyribonucleic acid synthesis and act as a chain terminator. ^20^^,^^21^

### Strengths of the case report

The study was conducted using face- to- face communication with the patient and was free from selection bias, response bias, and information bias. There is no feedback barrier among the investigator and respondent because the study was done through direct observational method. Relevant information was supported with pictures of the patient to show the spots on the patient’s body articulately. The study reported clinical manifestations, diagnosis and treatment of the patient starting from admission date to discharge date without any financial and time barriers.

### Limitations of the case report

One limitation was the inaccessibility of diagnostic equipment, especially an immunofluorescence assay which is more sensitive and reliable for the diagnosis of chickenpox infection. The numbers of rashes that was occurred on the body in successive days was counted manually. The patient refused to show the pictures of his face for fear of stigma. No follow up was performed after the patient was discharged whether cured totally and returned back to his usual skin tone or not. The study was not conducted based on systematic studies to distinguish the predictor’s factors of the chickenpox infection.

## Conclusion

Chickenpox is an infection caused by the varicella zoster virus; and characterized by itchy red blisters that appear almost all over the body. The main route of chickenpox infection transmission from person-to-person is through direct contact of open lesions or by inhalation of aerosolized droplets from respiratory tract secretions of patients with chickenpox. According to this case report there are three phases of chickenpox rash. I) Spots, and a tiny red rash that started on July 26/2022. II) Blisters, where spots in the first phase converted to fluid filled vesicles on July 27/2022. III) Crusts, in this phase the fluid filled vesicles that occurred in the blister phase dried out or converted to a hard rash. The fluid filled vesicles were counted in high numbers on the back, face, back and front of the neck and chest most commonly, and rarely counted on the buttocks and in the right outer ear. The patient was treated with calamine lotion, acetaminophen, cetirizine and acyclovir. For the management of chickenpox infection, oral acyclovir was given to the patient within 24 hours of the onset of the rash to treat the infection more effectively. Acyclovir prevents the duplication of the varicella zoster virus, and has the potential to eliminate varicella zoster virus.

## Data availability

All data underlying the results are available as part of the article and no additional source data are required.

## Consent

The author obtained the patient’s written informed consent to participate in this study and for the publication of images and data included in this case report.

## Author endorsement

Dr Subasini Uthirapathy confirms that the author has an appropriate level of expertise to conduct this research, and confirms that the submission is of an acceptable scientific standard. Dr Subasini Uthirapathy declares they have no competing interests. Affiliation: Tishk International University, Iraq.

## References

[1] Lança A Bernardo M Pinto S : Paediatric erythema multiforme: not every bullous rash is chickenpox. *BMJ Case Rep.* 2021;14:e246520. 10.1136/bcr-2021-246520 34969800 PMC8719153

[2] Blumental S : Management of varicella in neonates and infants/Sophie Blumental, Philippe Lepage. *BMJ Paediatrics Open. – 2019.* 2020;3(3):2720P. e000433. 10.1136/bmjpo-2019-000433 PMC6570487 31263790

[3] Boyd G Heaton PA Wilkinson R : Nursing management of childhood chickenpox infection. *Emerg. Nurse.* 2017;25:32–41. Date of submission: 1 April 2017; date of acceptance: 5 June 2017. 10.7748/en.2017.e1720 29219259

[4] Kujur A Kiran K Kujur M : An Epidemiological Study of Outbreak Investigation of Chickenpox in Remote Hamlets of a Tribal State in India. *Cureus.* June 30, 2022;14(6):e26454. 35923668 10.7759/cureus.26454 PMC9339339

[5] Parente S : Management of chickenpox in pregnant women: an Italian perspective. *Eur. J. Clin. Microbiol. Infect. Dis.* 2018;37:1603–1609. 10.1007/s10096-018-3286-7 29802481 PMC7101639

[6] Rodriguez-Santana Y Sanchez-Almeida E Garcia-Vera C : PAPenRED. Epidemiological and clinical characteristics and the approach to infant chickenpox in primary care. *Eur. J. Pediatr.* 2019;178:641–648. 10.1007/s00431-019-03332-9 30767142

[7] Riera-Montes M Bollaerts K Heininger U : Estimation of the burden of varicella in Europe before the introduction of universal childhood immunization. *BMC Infect. Dis.* 2017;17:353. 10.1186/s12879-017-2445-2 28521810 PMC5437534

[8] Mareschal A : Photodistributed chickenpox in a 3-year-old boy. *CMAJ.* 2021 March 22;193:E425. 10.1503/cmaj.201771 33753367 PMC8096382

[9] Habek M : Chickenpox and asymptomatic COVID-19 after first cycle of alemtuzumab for multiple sclerosis. *Neurol. Sci.* 2021;42:4003–4005. 10.1007/s10072-021-05495-6 34331616 PMC8325041

[10] Zoghaib S Kechichian E Souaid K : Triggers, clinical manifestations, and management of pediatric erythema multiforme: a systematic review. *J. Am. Acad. Dermatol.* 2019;81:813–822. 10.1016/j.jaad.2019.02.057 31331726

[11] Riera-Montes M Bollaerts K Heininger U : Estimation of the burden of varicella in Europe before the introduction of universal childhood immunization. *BMC Infect. Dis.* 2017;17(1):353. 10.1186/s12879-017-2445-2 28521810 PMC5437534

[12] Mikaeloff Y Kezouh A Suissa S : Nonsteroidal anti-inflammatory drug use and the risk of severe skin and soft tissue complications in patients with varicella or zoster disease. *Br. J. Clin. Pharmacol.* 2008;65(2):203–209. 10.1111/j.1365-2125.2007.02997.x 18251759 PMC2291221

[13] National Institute for Health and Clinical Excellence: Clinical Knowledge Summary: Chickenpox. 2016. (Last accessed: 12 January 2017.) Reference Source

[14] Cohen J : Chickenpox: treatment. *Clin. Evid.* 2015;06:912. PMC4468609 26077272

[15] Public Health England: Chapter 34. Varicella. 2015. (Last accessed: 12 July 2017.) Reference Source

[16] Joint Formulary Committee: *British National Formulary.* London: BMJ Group and Pharmaceutical Press;2016;72.

[17] Cameron JC : Severe complications of chickenpox in hospitalized children in the UK and Ireland. *Arch. Dis. Child.* 2007;92:1062–1066. 10.1136/adc.2007.123232 17991685 PMC2066097

[18] Quaglietta L : Serious infectious events and ibuprofen administration in pediatrics: a narrative review in the era of COVID-19 pandemic. *Ital. J. Pediatr.* 2021;47:20. 10.1186/s13052-021-00974-0 33514404 PMC7844800

[19] Bansod V : Overview of Chickenpox in Children. *Int. Res. J. Mod. Eng. Technol. Sci.* February-2021;03(02):222–223.

[20] Gilden D : Varicella zoster virus vasculopathies: diverse clinical manifestations, laboratory features, pathogenesis, and treatment. *Lancet Neurol.* 2009 August;8(8):731–740. 10.1016/S1474-4422(09)70134-6 19608099 PMC2814602

[21] Baljic R : Therapeutic Approach to Chickenpox in Children and Adults-our Experience. *Med. Arh.* 2012 Jun;66(3 suppl 1):21–23. 10.5455/medarh.2012.66.s21-s23 22937685

