# Case Report: A Case Report: Chickenpox Acquired Through Surface Contamination: A Rare Clincal Observation

**DOI:** 10.12688/f1000research.124959.4

**Published:** 2025-10-15

**Authors:** Gudisa Bereda

**Affiliations:** 1 Pharmacy, Negelle health science college, Negelle, Oromia regional state, 1000, Ethiopia

**Keywords:** Case reports, Chickenpox infections, Healthcare workers, Acyclovir, progression, Varicella-zoster virus

## Abstract

Chickenpox is an extremely contagious disease caused by a primary varicella-zoster virus (VZV) infection. This case report describes a rare transmission of chickenpox potentially linked to an indirect route via surface contamination rather than direct person-to-person contact. A 27-year-old adult Black African male health care worker presented with severe headache, intermittent weakness and inability to walk, intermittent nausea, fever, shortness of breath, itching, pruritus, lesions with pus on the skin, sleep disturbances, and nightmares for two days. In this case report, the patient’s face, neck, and scapulae were the most infected areas of the body, and the rest of the body, except the legs, hands, genital areas, and buttocks, was also highly infected. Chickenpox was confirmed by positive polymerase chain reaction (PCR), positive IgM, viral culture, and low IgG. Acyclovir 800 mg orally, five times a day, was given for ten days to treat chickenpox infection because acyclovir inhibits varicella zoster virus replication. It is typically unable to eradicate VZV from the latent state in neurons. The patient showed marked improvement within a week and fully recovered without complications or post-herpetic sequelae. This case emphasizes the importance of strict infection control and hygiene protocols in preventing rare indirect transmission of the VZV, particularly in healthcare settings.

## Introduction

Chickenpox is an extremely contagious disease that occurs as a result of a primary varicella-zoster virus infection ( Lança A et al., 2021; Blumental S, 2020). The chickenpox infection disease course can rarely be more severe than expected and can spread to involve different organs and cause severe complications ( Boyd G et al., 2017). In healthy individuals, chickenpox infection can often be a mild, self-limiting disease described by fever, malaise, and generalized itchy and vesicular rash ( Kujur A et al., 2022). When comparing children’s clinical manifestations of chickenpox with adults’; in adults they are more severe and more frequently associated with complications ( Parente S et al., 2018; Rodriguez-Santana Y et al., 2019; Riera-Montes M et al., 2017). The WHO estimates 140 million global cases of chickenpox annually ( Pan CX et al., 2022). Adult primary infections are rare (5–10%) but often more severe. In Ethiopia, limited vaccine availability leads to frequent outbreaks, mainly in children under 15 ( Tesema GA et al., 2020). Chickenpox progression can differ based on factors such as age, immune response, and vaccination status ( Kujur A et al., 2022; Rodriguez-Santana Y et al., 2019).

**Figure 3.**
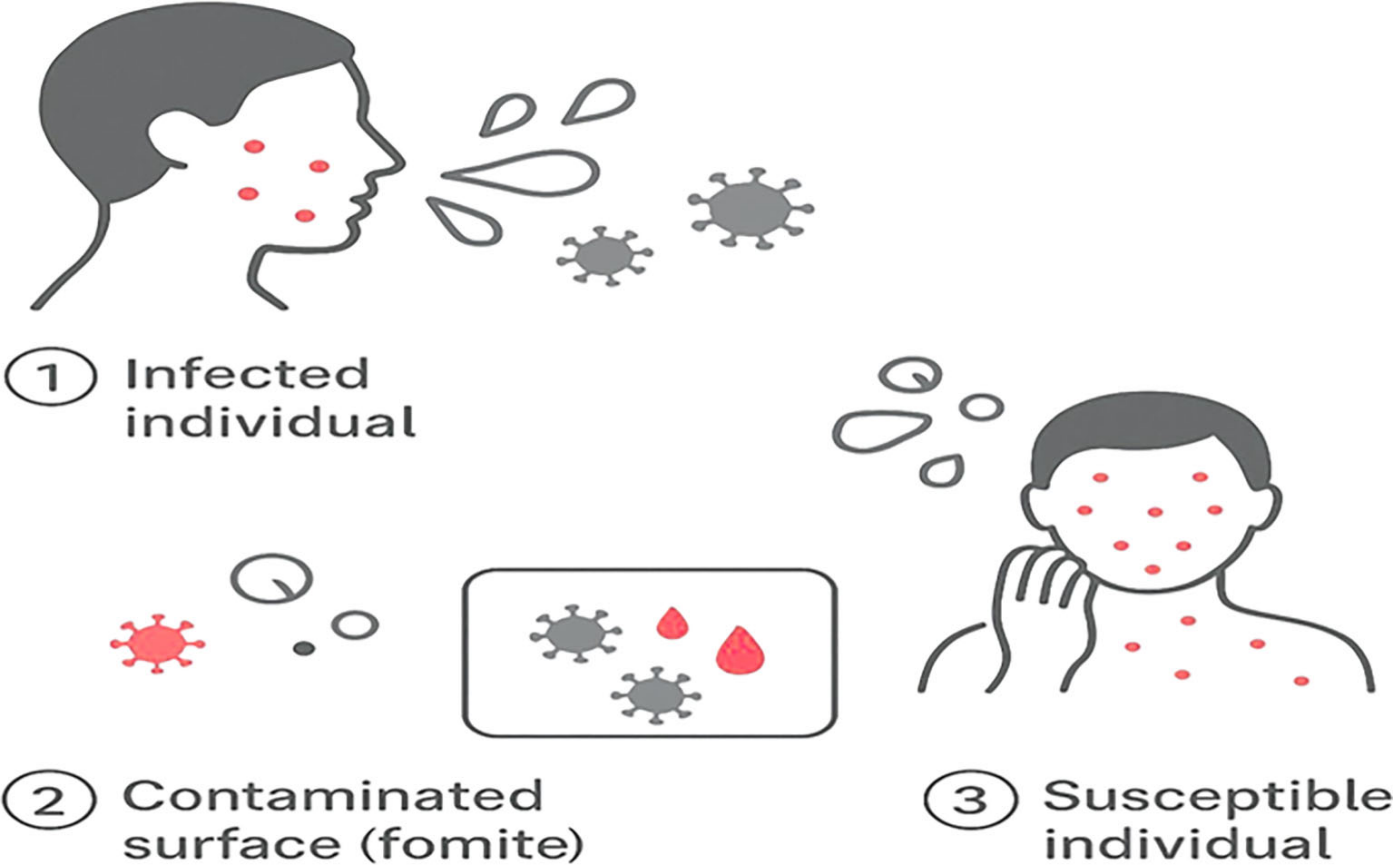
Schematic representation of the proposed indirect transmission pathway of chickenpox. (1) Infected individual: A contagious person sheds varicella-zoster virus (VZV) through respiratory droplets or vesicular fluid, releasing viral particles into the environment. (2) Contaminated surface (fomite): Viral droplets settle on nearby objects such as bedrails, clothing, or medical instruments, allowing VZV to persist for hours under favorable conditions. (3) Susceptible individual: A non-immune person contacts the contaminated surface and subsequently touches the contaminated surfaces or fomite, leading to self-inoculation and infection.

The rash of chickenpox infection is most frequently distributed over the trunk, scalp, and face ( Mareschal A et al., 2021). Chickenpox is spread by direct person-to-person contact with open lesions or airborne droplets and tends to increase in severity with each subsequent case within a household. It also transmitted by surfaces contaminated with the virus via blister fluid from an infected person ( Habek M, 2021; Riera-Montes M et al., 2017). Transmission via contaminated surfaces (fomites) is less commonly reported and considered a rare mode. The virus can survive on surfaces for several hours; however, the risk of infection through contaminated objects is generally lower than that of direct transmission ( Levin MJ et al., 2016). Laboratory confirmation such as PCR or serological testing may be employed in atypical or severe cases to support clinical diagnosis ( Hodinka RL, 2016). Acyclovir, a competitive inhibitor of viral DNA synthesis, acts as a chain terminator, but it cannot eradicate latent VZV from neurons. For adults, treatment of chickenpox infection with acyclovir was initiated at a dose of 800 mg orally five times a day for ten days ( Parente S et al., 2018; Boyd G et al., 2017). This case report demonstrates the clinical manifestations and sites of severe infections occurred in successive days until peeled off in an adult health care worker.

## What this study adds

This study provides comprehensive knowledge and new insights into the less common transmission of varicella zoster virus (VZV), which causes chickenpox, via contaminated surfaces. In this case report, VZV was spread by contact with surfaces contaminated with the virus via blister fluid from an infected person. The surface-contaminated route of transmission, when compared with direct contact with lesions and airborne distribution, is thought to be a less significant mechanism of transmission. This case occurs in a surprising age bracket, particularly in healthy adults who are not immunocompromised and have never had chickenpox.

## Case report

A 27-year-old adult Black African male healthcare worker was admitted on July 28, 2022. The admitted patient had no medical or medication history and no family medical or medication history. On admission, the patient presented with severe headache, intermittent weakness and inability to walk, intermittent nausea, fever, nocturnal polydipsia, shortness of breath, itching, pruritus (intensely pruritic erythematous macules), lesions with pus on the skin, sleep disturbances, and nightmares for two days. The erythematous pruritic macules converted into clear fluid-filled vesicles on the face after 12 hours and on other infected areas, such as the trunk, scalp, scapulae, back, and lower extremities in 24 hours. The admitted patient was taking a shower in their workplace at 5:00 pm with cold water and using soap and cloth in the shower room on July 25, 2022. Rare clinical manifestations such as fever and tiny lesions appeared on the face the morning after the shower. By the evening, lesions appeared on several sites on the body, especially around the scapulae, neck, and chest, and the number of lesions on the face had increased. He had no prior medical history of tuberculosis (TB), human immunodeficiency virus (HIV), hepatitis B or C, or any other immunocompromised diseases. Before the varicella zoster virus infection, he was in good health. Although he did not interact with the patient who had VZV, he showered with soap and dried his body with a cloth in the shower room. Throughout the assessment, his blood pressure was 121/81 mmHg, axillary temperature was 38.4 degrees Celsius, peripheral pulse rate was 78 beats/min, and respiration rate was 19 cycles/min. Blood tests revealed a hemoglobin was 15.1 mg/dl, white blood cell count of 10,540 cells/mm³, red blood cell count of 4.7 million/mm³, and neutrophil count of 59%. Blood urea nitrogen was 44 mg/dl, creatinine was 98 μmol/L, alkaline phosphatase was 215 U/L, gamma-glutamyl transferase was 19 U/L, and alanine transferase levels were and 9 IU/L. Liver and kidney function tests revealed no remarkable acute alterations. Chest radiography revealed no signs of community-acquired pneumonia. Examination of the heart, lungs, and abdomen revealed no noteworthy results.

The differential diagnosis included smallpox, drug eruptions, HFMD, herpes simplex, and disseminated herpes zoster. Herpes simplex causes grouped vesicles in specific areas, unlike the widespread rash of chickenpox. Drug eruptions may cause widespread rashes but differ in symptoms. The patient had no medication history or signs such as angioedema. Disseminated herpes zoster occurs in older or immunocompromised patients. HFMD affects the hands, feet, and mouth, not the whole body. Impetigo causes pustules without systemic symptoms. Chickenpox lesions appear in multiple stages, unlike smallpox. Mpox can mimic chickenpox with evolving vesicles and systemic symptoms.

Chickenpox was confirmed by positive VZV PCR, positive IgM, low IgG, and viral culture. Negative PCR and IgM excluded HSV and HFMD. Smallpox was ruled out clinically and by lab results. Drug eruptions were excluded due to lack of clinical signs, drug history, and biopsy findings. Disseminated herpes zoster was unlikely given negative PCR, rash pattern, and absence of IgG indicating no prior exposure. Additionally, a PCR test for mpox was conducted and returned negative, ruling out mpox infection ( Table 1).

**Table 1.** Diagnoses and tests performed to rule out other differential diagnoses.

Chickenpox differentials	Diagnosis and test performed	Results	Explanation
Chickenpox (VZV)	PCR of vesicular lesion swab	Positive	VZV DNA detected, confirming active viral infection
	VZV-specific IgM (Serology)	Positive (1:80 titer)	Indicates recent or acute VZV infection
	VZV-specific IgG (Serology)	Low titer (1:10)	Indicates no prior immunity or vaccination
	Viral culture from lesion	Positive	Live virus isolated, confirming active infection
Herpes Simplex Virus (HSV)	PCR of lesion swab	Negative	No HSV DNA detected, ruling out herpes simplex infection
	HSV IgM (Serology)	Negative	No evidence of recent HSV infection
	HSV viral culture	Negative	No HSV isolated from lesion
Hand, Foot, and Mouth Disease (HFMD)	PCR for Enteroviruses	Negative	No enterovirus RNA detected, excluding HFMD diagnosis
	Enterovirus IgM (Serology)	Negative	No recent enterovirus infection
Smallpox	Clinical evaluation	Not performed	Clinical presentation inconsistent; smallpox eradicated globally
	Smallpox IgM/IgG Serology	Not performed	Not performed as smallpox is eradicated; no clinical suspicion
Drug Eruption	Clinical history, skin biopsy	Negative	No drug exposure history; biopsy shows no drug-induced reaction
Disseminated Herpes Zoster	PCR for VZV	Low viral load	Absence of VZV reactivation typical markers
	VZV IgG (Serology)	Negative	Indicates no prior VZV exposure
	Clinical pattern assessment	Vesicular rash not dermatomal	Rash distribution inconsistent with herpes zoster

**Figure 1.**
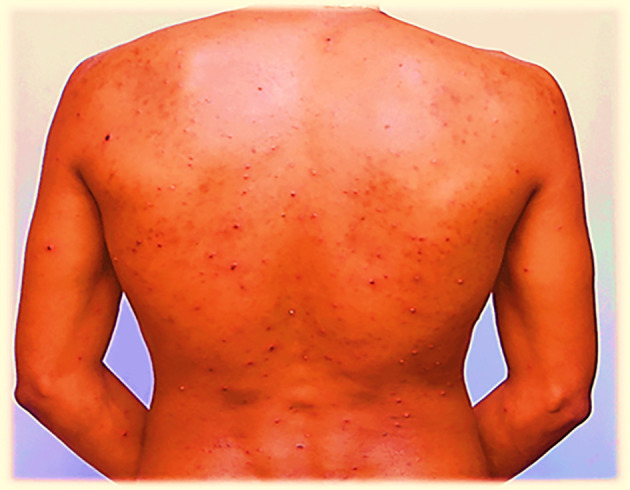
Red rash with ruptured lesions on the back, especially at the scapulae and lower back of the patient. This picture was captured at day three of the infection.

**Figure 2.**
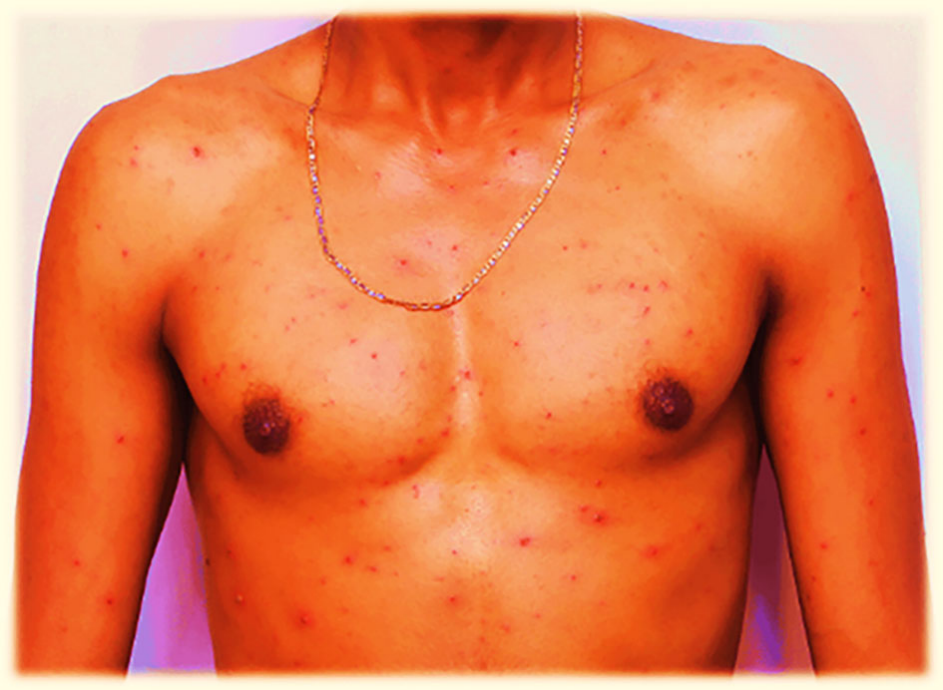
Red rash with ruptured lesions on the anterior body, especially on and around the chest of the patient. This picture was captured at day three of the infection.

On July 29, 2022, the lesions or maculae on the face transformed into fluid-filled vesicles, increased in number, and enlarged in size. They spread further on the body, especially to the scapulae and around the deltoid muscles, back and front of the neck, chest, and lower to the waist. The patient developed a severe headache and experienced itching over the entire body. Red rashes were especially prominent around the back and front of the neck, with fluid-filled vesicles. There was also a fluid-filled vesicle around the genital area and the buttock. Red rashes rarely occurred in the outer ear, with fluid-filled vesicles in and around the left outer ear and in the right outer ear. Red rashes developed on the scalp with fluid-filled vesicles. Given its extreme enlargement compared to other infected areas, the red rash on the face appeared to be more severe than that at other sites. The redness seen in Figures 1 and 2 also appears on the face.

On July 30, 2022, the fluid-filled vesicles on the face dried and converted to a hard rash (crusts), but on the upper and lower extremities, the fluid-filled vesicles remained the same on all the body with intermittent severe headache and itching. The number of red rashes on the back of the patient increased, and on the anterior side of the body, especially on and around the chest, fluid-filled vesicles increased. On August 01, 2022, the redness on the face had almost blackened and disappeared or changed to crusted or hard rashes, and the redness elsewhere on the body had enlarged in size and increased in number from July 30, 2022, and started to appear on the legs and hands in small amounts. On August 02, 2022, the red rash on the face completely peeled off, and the previous vesicles on the face changed to a black color rather than the usual skin tone. The redness elsewhere on the body, except the face and neck, slightly decreased in size and number but continued to spread on the legs and hands.

On August 03, 2022, the rash on the face completely disappeared, and where the vesicles had previously appeared, the skin blackened. The rash on the torso changed from a red rash to a black or crusted rash and started to decrease in size. On the hands and legs, the rash increased in size and was multiplied by the number of vesicles. On August 04, 2022, the rash on the face had completely disappeared, and the site of infection had turned black, whereas on the rest of the body, the rash sites had blackened except for the hands and legs. On August 05, 2022, the rash on the body completely dried and converted the infected areas to a blackened color; however, on the hands and legs, the rash had only partially changed to a black color. On August 06, 2022, the rashes on the hands and legs became crusted with a black color at the infected sites. Finally, on the morning of August 07, 2022, crusted rashes on the hands, legs, genital area, and buttocks peeled off and blackened the infected area.

The patient was treated with three medications starting from the date of admission. I) Acetaminophen 500 mg orally, three times a day for five days, was administered to alleviate fever or sores induced by chickenpox infection. II) Cetirizine hydrochloride (10 mg) was administered orally twice a day for five days to minimize itching and prevent the patient from scratching the rash and blisters, especially at night. III) Acyclovir 800 mg orally, five times a day for ten days, was administered to cure chickenpox infection because acyclovir inhibits the replication of the varicella zoster virus, eradicates varicella zoster virus, and relieves symptoms more readily. At discharge, a zinc calamine lotion of 8% calamine and 8% zinc was administered twice daily for five days to relieve itchiness and prevent further skin infections. Treatment began on day 2 and continued until day 11, leading to symptom improvement and complete healing by day 14.

### Follow-up and outcomes

Healthcare providers monitor signs to ensure that patients recover without any issues related to complications. He recommended taking the medication as directed by a physician and refraining from scratching the lesions to prevent bacterial infections. The blisters and rash had completely healed and had no lasting complications. Usually, in ten days his rash disappeared. He did not acquire dormant shingles, which are latent in neurons. He recovered without any skin infections, encephalitis, pneumonia, or neurological problems. After a 10-day follow-up period, the patient was sent home.

## Discussion

Chickenpox, caused by VZV, is a highly contagious disease mainly affecting children. Transmission through surface contamination is rare but plausible, as VZV can survive on fomites long enough to infect susceptible individuals ( Falcón DI et al., 2023) ( Figure 3). Respiratory droplets and direct contact are the main routes, but this case adds evidence that indirect transmission via contaminated surfaces can occur, especially where viral shedding is high and sanitation is poor ( La Rosa G et al., 2013). Despite limited surface viability, a high viral load in lesions may increase the risk of contamination. This case shows that chickenpox can be contracted indirectly without direct contact, emphasizing the importance of environmental hygiene. It also highlights the rarity and severity of adult chickenpox, especially in immunocompetent individuals.

Lesions swiftly develop within approximately 12 hours into 1 to 3 mm clear vesicles encircled by narrow red halos (“dew drops on a rose petal”) ( Saleh HM et al., 2025). Although the patient developed symptoms within 24 hours of using the public shower—shorter than the typical 10–21-day incubation period for chickenpox—rapid onset can occur under certain circumstances, such as high viral exposure or host susceptibility ( Lança A et al., 2021; Blumental S, 2020). No direct contact with a varicella-infected individual was reported at the workplace; therefore, airborne transmission from an asymptomatic or pre-symptomatic person cannot be entirely excluded. Transmission via contaminated surfaces, such as shower facilities, is theoretically possible but extremely rare and is considered a less likely route compared to airborne spread ( Levin MJ et al., 2016).

Chickenpox is frequently experienced by children, with a peak incidence in those aged less than 10 years, but it can infect any age group ( Zoghaib S et al., 2019; Rodriguez-Santana Y et al., 2019; Lança A et al., 2021). The most common symptom of chickenpox is a vesicular rash that appears on the scalp, face, and trunk and then disseminates distally to the limbs ( Riera-Montes M et al., 2017; Mikaeloff Y et al., 2008; Marin M et al., 2016). In this case report, the face, neck, and scapulae were the most infected areas of the body, and the rest, except the legs, hands, genital areas, and buttocks, were also highly infected. The legs, hands, genital areas, and buttocks did not exhibit as much infection, especially in the genital area, where only one red rash appeared. There are three phases of clinical manifestations of chickenpox: I) short prodromal phase, which appears 1-2 days after infection and comprises of mild or moderate fever. Red spots soon develop into blisters that are itchy and packed with fluid. II) The exanthematous phase, which appears for 3-5 days, comprises a rash that appears on day. The first rash transformed into clear fluid-filled vesicles and later converted into a hard rash that became crusted. In the exanthematous phase, the rash more commonly appears on the scapulae, chest, face, and above and around the lower back. Over the following few days, new blisters may form in waves, and previous blisters may begin to crust. III) The final phase, which appears for 6-10 days, is the convalescent phase or remedial phase, in which the crust usually resolves within seven days. Blisters continue to dry, crust, and eventually scab ( National Institute for Health and Clinical Excellence, 2016). Usually, chickenpox blisters or rashes persist for five–ten days. The rash or blisters of this patient persisted for 9 days, even though they decreased daily; finally, it disappeared in ten days. The patient did not develop shingles, which could arise from VZV latency in sensory neurons.

Key aspects of this report include the unusual pattern of VZV transmission, with the patient potentially contracting the virus from a contaminated surface in a shared shower room, highlighting the rare but significant risk of surface contact as a transmission route. Administering gamma globulin prophylactically to household contacts exposed to chickenpox could reduce disease severity and progression. This approach is particularly beneficial in immunocompromised individuals or those at risk of severe varicella complications, such as young children, pregnant women, or individuals with chronic conditions ( Presti CL et al., 2019; Ross AH, 1962; Mareschal A et al., 2021). While chickenpox spots develop over a few days, mpox spots emerge simultaneously. Generally, mpox lesions are deeper and larger than chickenpox lesions. While chickenpox blisters are itchy, mpox blisters can cause agony. Spots of the mpox simultaneously blister and crust. Crusty sores, blisters, and chickenpox patches, all at once. The VZV and MPXV (monkeypox virus) produce chickenpox and mpox respectively. The patient tested positive for VZV with both skin swabs and serology (IgG) antibodies. A negative PCR for mpox ruled out concurrent mpox infection ( Rasizadeh R et al., 2023; Class MM et al., 2024). The main goal in the management of chickenpox infection is to alleviate symptoms, such as skin infections, fever, and itching, and to make the individual comfortable ( Cohen J et al., 2015; Public Health England, 2015). Calamine lotions have skin-soothing properties and can be used to relieve itching ( Joint Formulary Committee, 2016). Since calamine lotion reduces severe itching, stops skin infections, and helps dry off leaking skin, it is frequently used to treat chickenpox. Zinc oxide in the calamine lotion serves as an antiseptic, which helps cleanse the skin and remove bacteria ( Sharma P et al., 2022). Acetaminophen is the preferred painkiller for the management of chickenpox associated with fever because of its very rare risk of non-steroidal anti-inflammatory drug-induced skin blisters and rashes ( Cameron JC et al., 2007; Quaglietta L et al., 2021). Cetirizine can alleviate itching and inhibit excoriation. Acyclovir prevents replication of varicella-zoster virus. Acyclovir 800 mg, administered orally five times a day for 10 days was used to treat chickenpox infection because it prevents the varicella zoster virus from replicating ( Bansod V et al., 2021). In this case report, acyclovir was effective because it was recommended for adolescents, especially those aged ≥ 12 years. Acyclovir can sometimes eradicate varicella zoster virus, but it can also be resistant to some strains of the virus. Acyclovir triphosphate is a competitive inhibitor of viral deoxyribonucleic acid synthesis and acts as a chain terminator ( Gilden D et al., 2009; Baljic R et al., 2012; Mareschal A et al., 2021). After 7–10 days of acyclovir treatment, persistent lesions were considered resistant to VZV. Its latency is established in human sensory neurons, specifically in the cranial and dorsal root ganglia, where it remains dormant. Acyclovir is typically unable to eradicate VZV from the latent state in neurons ( Kennedy PG et al., 1998).

### Strengths of the case report

The study was conducted using face-to-face communication with the patients and was free from selection bias, response bias, and information bias. There was no feedback barrier between the investigator and the respondent because the study was conducted using a direct observational method. Relevant information was supported by pictures of the patient to articulate the spots on the patient’s body. The study reported the clinical manifestations, diagnosis, and treatment of the patient from the admission date to the discharge date without any financial or time barriers.

### Limitations of the case report

One limitation is the inaccessibility of diagnostic equipment, especially an immunofluorescence assay, which is more sensitive and reliable for the diagnosis of chickenpox infection. The patient refused to show pictures of his face because of fear of stigma. No follow-up was performed after the patient was discharged, whether cured completely (returned to his usual skin tone) or not. This study was not conducted based on systematic studies to identify the predictors of chickenpox infection.

### How this study might be useful for other practitioners

A more thorough examination of patient risk variables will help clinicians to identify patients, particularly young adults, who may be more susceptible to problems. They provided supportive care such as controlling pruritus, managing fever, and taking care of the skin to avoid secondary infections. It included comprehensive instructions on how to avoid and treat complications, such as encephalitis, pneumonia, and bacterial infections, which could help medical professionals provide preventative care.

## Conclusion

Chickenpox is an infection caused by varicella zoster virus and is characterized by itchy red blisters that appear almost all over the body. In this case report, VZV was spread by contact with surfaces contaminated with the virus via blister fluid from an infected person. According to this case report, there are three phases of chickenpox rash. I) Spots and a tiny red rash that started on July 26, 2022. II) Blisters, where spots in the first phase were converted to fluid-filled vesicles on July 27, 2022. III) Crusts: In this phase, fluid-filled vesicles that occurred in the blister phase dried out or converted to a hard rash. Fluid-filled vesicles were observed in high numbers on the back, face, and back and front of the neck and chest, and were rarely counted on the buttocks and in the right outer ear. To manage chickenpox infection, oral acyclovir was administered to the patient within 24 hours of the onset of the rash to treat the infection more effectively. Acyclovir prevents the replication of varicella zoster virus and has the potential to eliminate varicella zoster virus. It is typically unable to eradicate VZV from the latent state in neurons.

## Data availability

All data underlying the results are available as part of the article and no additional source data are required.

## Consent

The author obtained written informed consent from the patient to participate in this study and for the publication of images and data included in this case report.

## Author endorsement

Dr. Subasini Uthirapathy confirms that the author has an appropriate level of expertise to conduct this research and that the submission is an acceptable scientific standard. Dr. Subasini Uthirapathy declares that they have no competing interests. Affiliations: Tishk International University, Iraq. Dr. Uthirapathy provided expert supervisory support throughout the preparation and thorough review of the case reports, ensuring accuracy, clarity, and adherence to ethical and clinical standards.

## References

[ref1] Baljic R : Therapeutic Approach to Chickenpox in Children and Adults-our Experience. *Med. Arh.* 2012Jun;66(3 suppl1):21–23. 10.5455/medarh.2012.66.s21-s23 22937685

[ref2] Bansod V : Overview of Chickenpox in Children. *Int. Res. J. Mod. Eng. Technol. Sci.* 2021;03(02):222–223.

[ref3] Blumental S : Management of varicella in neonates and infants/Sophie Blumental, Philippe Lepage. *BMJ Paediatr. Open.* 2019;3(3):e000433. 10.1136/bmjpo-2019-000433 31263790 PMC6570487

[ref4] Boyd G Heaton PA Wilkinson R : Nursing management of childhood chickenpox infection. *Emerg. Nurse.* 2017;25:32–41. Date of submission: 1 April 2017; date of acceptance: 5 June 2017. 10.7748/en.2017.e1720 29219259

[ref5] Cameron JC : Severe complications of chickenpox in hospitalized children in the UK and Ireland. *Arch. Dis. Child.* 2007;92:1062–1066. 10.1136/adc.2007.123232 17991685 PMC2066097

[ref6] Class MM McCoy K Hafeez F : Case Letter. *Cutis.* 2024Oct;114(4):E29–E31. 10.12788/cutis.1133 39621563

[ref7] Cohen J : Chickenpox: treatment. *Clin. Evid.* 2015;06:912. PMC4468609 26077272

[ref28] Falcón DI Báez DA Vera MD : Varicella Zoster Virus (VZV) Infection: A Comprehensive Review of Chickenpox. *Public Health.* 2023 Oct;27(1):2.

[ref8] Gilden D : Varicella zoster virus vasculopathies: diverse clinical manifestations, laboratory features, pathogenesis, and treatment. *Lancet Neurol.* 2009 August;8(8):731–740. 10.1016/S1474-4422(09)70134-6 19608099 PMC2814602

[ref9] Habek M : Chickenpox and asymptomatic COVID-19 after first cycle of alemtuzumab for multiple sclerosis. *Neurol. Sci.* 2021;42:4003–4005. 10.1007/s10072-021-05495-6 34331616 PMC8325041

[ref29] Hodinka RL : Serologic tests in clinical virology. InLennette’s Laboratory Diagnosis of Viral Infections. 2016 Apr 19; pp.133–150.

[ref10] Joint Formulary Committee : *British National Formulary.* London: BMJ Group and Pharmaceutical Press:2016;72.

[ref11] Kennedy PG Grinfeld E Gow JW : Latent varicella-zoster virus is located predominantly in neurons in human trigeminal ganglia. *Proc. Natl. Acad. Sci. U S A.* 1998 Apr 14;95(8):4658–4662. 10.1073/pnas.95.8.4658 9539794 PMC22546

[ref12] Kujur A Kiran K Kujur M : An Epidemiological Study of Outbreak Investigation of Chickenpox in Remote Hamlets of a Tribal State in India. *Cureus.* June 30, 2022;14(6): e26454. 10.7759/cureus.26454 PMC9339339 35923668

[ref13] Lança A Bernardo M Pinto S : Paediatric erythema multiforme: not every bullous rash is chickenpox. *BMJ Case Rep.* 2021;14: e246520. 10.1136/bcr-2021-246520 PMC8719153 34969800

[ref30] La Rosa G Fratini M Libera SD : Viral infections acquired indoors through airborne, droplet or contact transmission. *Ann. Ist Super Sanita.* 2013;49:124–132. 10.4415/ANN_13_02_03 23771256

[ref31] Levin MJ Weinberg A Schmid DS : Herpes simplex virus and varicella-zoster virus. *Diagn. Microbiol. Immunocompr. Host.* 2016 Aug;15:135–156. 10.1128/9781555819040.ch6 27337486

[ref14] Mareschal A : Photodistributed chickenpox in a 3-year-old boy. *CMAJ.* 2021 March 22;193:E425. 10.1503/cmaj.201771 33753367 PMC8096382

[ref15] Marin M Marti M Kambhampati A : Global varicella vaccine effectiveness: A meta-analysis. *Pediatrics.* 2016;137(3): e20153741. 10.1542/peds.2015-3741 26908671

[ref16] Mikaeloff Y Kezouh A Suissa S : Nonsteroidal anti-inflammatory drug use and the risk of severe skin and soft tissue complications in patients with varicella or zoster disease. *Br. J. Clin. Pharmacol.* 2008;65(2):203–209. 10.1111/j.1365-2125.2007.02997.x 18251759 PMC2291221

[ref17] National Institute for Health and Clinical Excellence : Clinical Knowledge Summary: Chickenpox.2016. (Last accessed: 12 January 2017).

[ref32] Pan CX Lee MS Nambudiri VE : Global herpes zoster incidence, burden of disease, and vaccine availability: a narrative review. *Ther. Adv. Vaccines Immunother.* 2022 Mar;10:25151355221084535. 10.1177/25151355221084535 35340552 PMC8941701

[ref18] Parente S : Management of chickenpox in pregnant women: an Italian perspective. *Eur. J. Clin. Microbiol. Infect. Dis.* 2018;37:1603–1609. 10.1007/s10096-018-3286-7 29802481 PMC7101639

[ref19] Presti CL Curti C Montana M Chickenpox: an update. *Médecine et Maladies Infectieuses.* 2019 Feb 1;49(1):1–8. 10.1016/j.medmal.2018.04.395 29789159

[ref20] Public Health England : Chapter 34. Varicella.2015. (Last accessed: 12 July 2017).

[ref21] Quaglietta L : Serious infectious events and ibuprofen administration in pediatrics: a narrative review in the era of COVID-19 pandemic. *Ital. J. Pediatr.* 2021;47:20. 10.1186/s13052-021-00974-0 33514404 PMC7844800

[ref22] Rasizadeh R Shamekh A Shiri Aghbash P : Comparison of human monkeypox, chickenpox, and smallpox: A comprehensive review of pathology and dermatological manifestations. *Curr. Med. Res. Opin.* 2023;39(5):751–760. 10.1080/03007995.2023.2200122 37025009

[ref23] Riera-Montes M Bollaerts K Heininger U : Estimation of the burden of varicella in Europe before the introduction of universal childhood immunization. *BMC Infect. Dis.* 2017;17:353. 10.1186/s12879-017-2445-2 28521810 PMC5437534

[ref24] Rodriguez-Santana Y Sanchez-Almeida E Garcia-Vera C : PAPenRED. Epidemiological and clinical characteristics and the approach to infant chickenpox in primary care. *Eur. J. Pediatr.* 2019;178:641–648. 10.1007/s00431-019-03332-9 30767142

[ref25] Ross AH : Modification of chicken pox in family contacts by administration of gamma globulin. *N. Engl. J. Med.* 1962;267:369–376. 10.1056/NEJM196208232670801 14494142

[ref40] Saleh HM Ayoade F Kumar S : Varicella-Zoster Virus (Chickenpox) [Updated 2025 Apr 27].In: StatPearls [Internet]. Treasure Island (FL): StatPearls Publishing;2025 Jan. Reference Source 28846365

[ref26] Sharma P Hasan MR Mehto NK : 92 years of zinc oxide: has been studied by the scientific community since the 1930s—an overview. *Sens. Int.* 2022;3:100182. 10.1016/j.sintl.2022.100182

[ref33] Tesema GA Tessema ZT Tamirat KS : Complete basic childhood vaccination and associated factors among children aged 12–23 months in East Africa: a multilevel analysis of recent demographic and health surveys. *BMC Public Health.* 2020 Dec;20:1–4. 10.1186/s12889-020-09965-y 33256701 PMC7708214

[ref27] Zoghaib S Kechichian E Souaid K : Triggers, clinical manifestations, and management of pediatric erythema multiforme: a systematic review. *J. Am. Acad. Dermatol.* 2019;81:813–822. 10.1016/j.jaad.2019.02.057 31331726

