# Supplementary material for: Case Report: A Case Report: Chickenpox Acquired Through Surface Contamination: A Rare Clincal Observation
Source: F1000Res. 2025 Oct 15;11:1004. Originally published 2022 Sep 6. [Version 4] doi: 10.12688/f1000research.124959.4 (PMC12592853; doi:10.12688/f1000research.124959.4)
Supplement: Peer review history

# Reviewer report: Reviewer response for version 4

**DOI:** 10.5256/f1000research.187832.r426587

**Published:** 2025-11-06

**Authors:** James Andrawus

**Affiliations:** 1 Federal University Dutse, Dutse, Jigawa, Nigeria

The background of hickenpox has been discussed well in this report.  Enough details were provided for physical examination and diagnostic tests, treatment given and outcomes. Sufficient discussion was included on the importance of the findings and their relevance to future understanding of disease processes, diagnosis or treatment. The case presented has adequate detail that can be useful to other practitioners.

Are enough details provided of any physical examination and diagnostic tests, treatment given and outcomes?

Yes

Is the case presented with sufficient detail to be useful for other practitioners?

Yes

Is sufficient discussion included of the importance of the findings and their relevance to future understanding of disease processes, diagnosis or treatment?

Yes

Is the background of the case’s history and progression described in sufficient detail?

Yes

Reviewer Expertise:

Mathematical Epidemiology

I confirm that I have read this submission and believe that I have an appropriate level of expertise to confirm that it is of an acceptable scientific standard.

# Reviewer report: Reviewer response for version 4

**DOI:** 10.5256/f1000research.187832.r424455

**Published:** 2025-11-06

**Authors:** Kathrin Sutter

**Affiliations:** 1 University of Duisburg-Essen, Essen, Germany

I am satisfied with the authors revision. They addressed all my concerns.

Are enough details provided of any physical examination and diagnostic tests, treatment given and outcomes?

No

Is the case presented with sufficient detail to be useful for other practitioners?

No

Is sufficient discussion included of the importance of the findings and their relevance to future understanding of disease processes, diagnosis or treatment?

Partly

Is the background of the case’s history and progression described in sufficient detail?

Yes

Reviewer Expertise:

infectiology, immunology, virology

I confirm that I have read this submission and believe that I have an appropriate level of expertise to confirm that it is of an acceptable scientific standard.

# Reviewer report: Reviewer response for version 3

**DOI:** 10.5256/f1000research.182839.r401722

**Published:** 2025-08-26

**Authors:** Theresa Paul

**Affiliations:** 1 Montefiore Wakefield Campus (Ringgold ID: 14841), New York, New York, USA; 2 Medicine, Hamad Medical Corporation, Doha, Qatar

The incubation period for chickenpox is usually between 10 to 21 days, most often around two weeks. So, it’s very unlikely that the patient developed symptoms within 24 hours of using the public shower—it doesn’t fit the expected timeline for infection.

 At the same time, even though there was no known direct contact at work, chickenpox spreads through the air, and people can be contagious even before they show any signs of illness. That makes it hard to completely rule out workplace exposure, especially from someone in the early stages of infection.

 As for transmission through surfaces like in a shower facility—chickenpox doesn’t survive well outside the body. While it’s not impossible, fomite transmission is extremely rare and not considered a significant route. That’s why linking this case to a shower exposure is questionable and less likely than airborne spread in a shared space.

Are enough details provided of any physical examination and diagnostic tests, treatment given and outcomes?

Yes

Is the case presented with sufficient detail to be useful for other practitioners?

No

Is sufficient discussion included of the importance of the findings and their relevance to future understanding of disease processes, diagnosis or treatment?

Yes

Is the background of the case’s history and progression described in sufficient detail?

Yes

Reviewer Expertise:

Internal medicine

I confirm that I have read this submission and believe that I have an appropriate level of expertise to state that I do not consider it to be of an acceptable scientific standard, for reasons outlined above.

# Response

**Published:** 2025-09-06

**Authors:** Gudisa Tola

**Affiliations:** negelle health science college, Ethiopia

Reviewer Comment 1: The incubation period for chickenpox is usually between 10 to 21 days, most often around two weeks. So, it’s very unlikely that the patient developed symptoms within 24 hours of using the public shower—it doesn’t fit the expected timeline for infection.

 Author Response: The author acknowledges that the typical incubation period for varicella-zoster virus ranges from 10 to 21 days. However, in this case, the early onset of symptoms may reflect either prior unnoticed exposure or an unusually rapid viral replication. While uncommon, accelerated presentations have been reported in certain adult cases, which we have highlighted in the discussion. Lesions swiftly develop within approximately 12 hours into 1 to 3 mm clear vesicles encircled by narrow red halos (“dew drops on a rose petal”) [1]. Saleh HM, Ayoade F, Kumar S. Varicella-Zoster Virus (Chickenpox) [Updated 2025 Apr 27]. In: StatPearls [Internet]. Treasure Island (FL): StatPearls Publishing; 2025 Jan-. Available from: https://www.ncbi.nlm.nih.gov/books/NBK448191/

 Reviewer Comment 2: Even though there was no known direct contact at work, chickenpox spreads through the air, and people can be contagious even before they show any signs of illness. That makes it hard to completely rule out workplace exposure, especially from someone in the early stages of infection.

 Author Response: The author agrees that airborne transmission in the workplace cannot be completely excluded. The patient may have been exposed to an asymptomatic or pre-symptomatic individual, and we have added this consideration to the discussion to acknowledge the limitations in tracing the exact source of infection.

 Reviewer Comment 3: As for transmission through surfaces like in a shower facility—chickenpox doesn’t survive well outside the body. While it’s not impossible, fomite transmission is extremely rare and not considered a significant route. That’s why linking this case to a shower exposure is questionable and less likely than airborne spread in a shared space.

 Author Response: The author concurs that fomite transmission of varicella-zoster virus is extremely rare and typically not a primary route. Nevertheless, given the temporal association with shower use, we have presented surface exposure as a possible, albeit unlikely, indirect transmission route, while emphasizing that airborne spread remains the more probable mode of infection.

# Reviewer report: Reviewer response for version 2

**DOI:** 10.5256/f1000research.177401.r377708

**Published:** 2025-05-24

**Authors:** Kathrin Sutter

**Affiliations:** 1 University of Duisburg-Essen, Essen, Germany

The case report by Gudisa Bereda describes a 27-year-old male who contracted chickenpox, allegedly through surface contamination. The clinical progression is presented in detail, with a thorough description of the symptoms experienced by the patient. However, the report lacks essential information regarding diagnostic testing. Specifically, results of important differential diagnoses, such as herpes simplex virus (HSV) and hand, foot, and mouth disease (HFMD), are not included, and the diagnostic methods as well as used tests themselves are insufficiently explained.

 The abstract provides only a superficial overview, listing symptoms and stating that the patient was treated with acyclovir. However, it fails to include any final outcomes, or conclusions, which significantly weakens its value and informative function.

 The author claim that this revised version of the case report highlights an unusual presentation of chickenpox in an unexpected age group, and that the mode of transmission—via surface contamination—is rare. Unfortunately, these aspects are neither adequately emphasized in the abstract nor supported by the main text. The introduction already acknowledges that varicella-zoster virus can be transmitted through surface contamination, which diminishes the novelty of this case. If the age-related rarity of the infection is indeed a central point, it should be more explicitly elaborated upon in the introduction and supported by relevant epidemiological data.

 The included images are difficult to interpret due to low resolution and insufficient magnification. Higher quality images with clearer labeling would significantly improve the report’s value. Additionally, incorporating the treatment administration into a timeline that would provide a clearer overview for the reader.

 Finally, the author endorsement mentions Dr. Uthirapathy from Iraq, while the case itself is from Ethiopia. The relevance of this endorsement to the case is unclear and should be clarified or omitted to maintain focus and coherence.

Are enough details provided of any physical examination and diagnostic tests, treatment given and outcomes?

No

Is the case presented with sufficient detail to be useful for other practitioners?

No

Is sufficient discussion included of the importance of the findings and their relevance to future understanding of disease processes, diagnosis or treatment?

Partly

Is the background of the case’s history and progression described in sufficient detail?

Yes

Reviewer Expertise:

Viral immunology, immunotherapy

I confirm that I have read this submission and believe that I have an appropriate level of expertise to state that I do not consider it to be of an acceptable scientific standard, for reasons outlined above.

# Response

**Published:** 2025-05-25

**Authors:** Gudisa Tola

**Affiliations:** negelle health science college, Ethiopia

Author response

 Q1) However, the report lacks essential information regarding diagnostic testing. Specifically, results of important differential diagnoses, such as herpes simplex virus (HSV) and hand, foot, and mouth disease (HFMD), are not included, and the diagnostic methods as well as used tests themselves are insufficiently explained. Author response: I have added a table showing the results of VZV testing and all relevant differential diagnoses. Chickenpox was confirmed by positive VZV PCR, positive IgM, low IgG, and viral culture. HSV and HFMD were excluded by negative PCR and negative IgM. Smallpox was ruled out clinically and by laboratory results. Drug eruptions were excluded based on the absence of clinical signs, lack of relevant drug history, and biopsy findings. Disseminated herpes zoster was deemed unlikely due to a negative PCR result, atypical rash distribution, and absence of IgG, indicating no prior exposure. Additionally, Mpox was ruled out by a negative PCR test result.

 Q2) The abstract provides only a superficial overview, listing symptoms and stating that the patient was treated with acyclovir. However, it fails to include any final outcomes, or conclusions, which significantly weakens its value and informative function Author response: I have revised the abstract to enhance clarity and completeness. This case report describes a rare transmission of chickenpox, potentially linked to an indirect route via surface contamination rather than direct person-to-person contact. The patient showed marked improvement within a week and fully recovered without complications or post-herpetic sequelae. Chickenpox was confirmed by positive polymerase chain reaction (PCR), positive IgM, viral culture, and low IgG. This case emphasizes the importance of strict infection control and hygiene protocols in preventing rare indirect transmission of the varicella-zoster virus (VZV), particularly in healthcare settings.

 Q3) The author claim that this revised version of the case report highlights an unusual presentation of chickenpox in an unexpected age group, and that the mode of transmission—via surface contamination—is rare. Unfortunately, these aspects are neither adequately emphasized in the abstract nor supported by the main text. The introduction already acknowledges that varicella-zoster virus can be transmitted through surface contamination, which diminishes the novelty of this case. If the age-related rarity of the infection is indeed a central point, it should be more explicitly elaborated upon in the introduction and supported by relevant epidemiological data. Author response: I have revised both the introduction and discussion sections to better emphasize the rarity of transmission via surface contamination and the unusual occurrence of adult-onset chickenpox in immunocompetent individuals. Introduction: Transmission via contaminated surfaces (fomites) is rarely reported. Although VZV can survive on surfaces for several hours, the risk of infection via fomites is significantly lower than direct transmission. (Levin MJ et al., 2016)  Discussion: While respiratory droplets and direct contact remain the primary transmission routes for VZV (Falcón DI et al., 2023), this case supports the plausibility of fomite-based transmission. VZV can remain viable on surfaces long enough to infect susceptible hosts, particularly when viral shedding is high and sanitation is suboptimal. (La Rosa G et al., 2013) This report underscores the potential for indirect transmission and the clinical significance of adult chickenpox, a condition that is rare but often more severe in immunocompetent adults."

 Q4) The included images are difficult to interpret due to low resolution and insufficient magnification. Higher quality images with clearer labeling would significantly improve the report’s value. Additionally, incorporating the treatment administration into a timeline that would provide a clearer overview for the reader. Author response: The images were captured at the time using a standard camera, and unfortunately, no higher-resolution versions are available now. However, to clarify the treatment course, I have included a treatment timeline: Day 2: Initiation of antiviral therapy (acyclovir). Days 3–11: Continued treatment. Day 14: Full recovery with complete lesion resolution

 Q5) Finally, the author endorsement mentions Dr. Uthirapathy from Iraq, while the case itself is from Ethiopia. The relevance of this endorsement to the case is unclear and should be clarified or omitted to maintain focus and coherence. Author response: Dr. Uthirapathy, although based in Iraq, provided expert supervisory support throughout the preparation and review of this case report. Her contributions ensured the report’s accuracy, clarity, and adherence to both clinical and ethical standards.

# Reviewer report: Reviewer response for version 2

**DOI:** 10.5256/f1000research.177401.r367292

**Published:** 2025-03-06

**Authors:** Charles Grose

**Affiliations:** 1 The University of Iowa, Iowa City, Iowa, USA

I am pleased with the responses to my comments. The revised manuscript is much improved.

Are enough details provided of any physical examination and diagnostic tests, treatment given and outcomes?

No

Is the case presented with sufficient detail to be useful for other practitioners?

No

Is sufficient discussion included of the importance of the findings and their relevance to future understanding of disease processes, diagnosis or treatment?

No

Is the background of the case’s history and progression described in sufficient detail?

Yes

Reviewer Expertise:

Expertise in both clinical and basic virology, especially of the human herpesviruses, such as varicella and herpes zoster. Expertise in pediatric infectious diseases.

I confirm that I have read this submission and believe that I have an appropriate level of expertise to confirm that it is of an acceptable scientific standard.

# Reviewer report: Reviewer response for version 1

**DOI:** 10.5256/f1000research.137210.r239358

**Published:** 2024-05-11

**Authors:** Charles Grose

**Affiliations:** 1 The University of Iowa, Iowa City, Iowa, USA

This case report in 2024 requires at least one diagnostic test to confirm the presence of varicella virus. The test does not need to be the most sophisticated test. Further, there are now several reports that demonstrate that mpox (monkeypox) can exhibit a virtually identical rash to that of varicella. There are also reports that show some patients can have a dual infection with both varicella and mpox. These patients with dual infection often have a more severe or prolonged rash. Therefore, tests for both varicella and mpox are needed.

 There are many older reports that have counted the number of skin vesicles in a typical skin rash of children and young adults with varicella. Therefore, counting the number of vesicles in a rash does not provide additional insight into the disease of varicella in 2024. Here is an old varicella reference in one of the most respected medical journals in the world: [ ref 1 ]. This article should be cited and discussed.

Are enough details provided of any physical examination and diagnostic tests, treatment given and outcomes?

No

Is the case presented with sufficient detail to be useful for other practitioners?

No

Is sufficient discussion included of the importance of the findings and their relevance to future understanding of disease processes, diagnosis or treatment?

No

Is the background of the case’s history and progression described in sufficient detail?

Yes

Reviewer Expertise:

Expertise in both clinical and basic virology, especially of the human herpesviruses, such as varicella and herpes zoster. Expertise in pediatric infectious diseases.

I confirm that I have read this submission and believe that I have an appropriate level of expertise to state that I do not consider it to be of an acceptable scientific standard, for reasons outlined above.

## References

[1] : Modification of chicken pox in family contacts by administration of gamma globulin. *N Engl J Med* .1962;267: 10.1056/NEJM196208232670801 369-76 10.1056/NEJM196208232670801 14494142

# Response

**Published:** 2024-11-17

**Authors:** Gudisa Tola

**Affiliations:** negelle health science college, Ethiopia

Reviewer 2

 Usually, chickenpox blisters or rash persist for five to ten days. The broad timeline is shown here. Day 1-2: Red spots that soon develop into blisters that are itchy and packed with fluid occur. Day 3–5: Over the following few days, new blisters may form in waves, and previous blisters may start to crust over. Day 6-10: Blisters continue to dry and crust, eventually scabbing over [1]. The rash or blisters of this patient stayed for 9 days, even though they decreased day to day; finally, it disappeared in ten days. 

 He had no prior medical history of tuberculosis (TB), human immunodeficiency virus (HIV), Hepatitis B or C, or any other immunocompromised diseases. Before getting the varicella zoster virus, he was in good health. Although he didn’t interact with the patient who had VZV, he showered with soap and dried his body with a cloth, which was in the shower room.

 What this study adds

 This study provides comprehensive knowledge and insights about less common transmission of the varicella zoster virus (VZV), which causes chickenpox, via contaminated surfaces. In this case report, VZV was spread by coming into contact with surfaces contaminated with the virus via blister fluid from an infected person. Surface-contaminated route of transmission, when compared with direct contact with lesions and airborne distribution, was thought to be a less significant mechanism of transmission. This instance is occurring in a surprising age bracket, particularly in healthy adults who are not immunocompromised and have never had chickenpox.

 How this study might be useful for other practitioners

 A more thorough examination of patient risk variables will help clinicians identify patients, particularly young adults, who may be more susceptible to problems. How they provided supportive care, such as controlling pruritus, managing fever, and taking care of the skin to avoid secondary infections. It included comprehensive instructions on how to avoid and treat complications like encephalitis, pneumonia, and bacterial infections, which could help medical professionals provide preventative care.

 Physical examinations

 Throughout the assessment, his blood pressure was 121/81 mmhg, his axillary temperature was 38.4 degrees Celsius, his peripheral pulse rate was 78 beats per minute, and his respiration rate was 19 cycles per minute. His blood tests revealed that his hemoglobin was 15.1 mg/dl, his white blood cell count was 10540 cells/mm3, his red blood cell count was 4.7 million/mm, and his neutrophil count was 59%. Blood urea nitrogen was 44 mg/dl, creatinine was 98 μmol/L, alkaline phosphatase was 215 U/L, gamma glutamyl transferase was 19 U/L, and alanine transferase was 9 IU/L. His liver and kidney function tests showed no appreciable acute alterations.

 The x-ray of his chest showed no signs of community-acquired pneumonia. Examinations of his heart, lungs, and abdomen revealed no noteworthy results. Results from polymerase chain reaction tests for VZV or skin lesions (a vesicle or scab material) were positive. He tested positive for VZV with both his skin swab and serology (IgG) antibodies. Serology (IgM) antibodies were positive, indicating a high level of recent VZV infection.

 Differential diagnosis

 In this case report, the differential diagnosis of chickenpox includes smallpox, drug eruptions, hand, foot, and mouth disease, herpes simplex virus, and disseminated herpes zoster. Grouped vesicles, particularly on the lips or vaginal area, are caused by the herpes simplex virus. As opposed to being ubiquitous like chickenpox, it typically affects specific locations. Although drug eruptions frequently feature mucosal lesions and systemic symptoms different from chickenpox, they can also result in a widespread vesicular or bullous rash. There was no sign of angioedema, skin necrosis, or erosions of the mucosal membranes in the patient. Before the rash appeared, the patient had not started taking any medicines.

 Disseminated herpes zoster, also known as shingles, is a reactivation of the varicella zoster virus that does not initial infection in chickenpox and occurs in older or immunocompromised patients. Hand, foot, and mouth disease (HFMD), as opposed to a broad body rash, is characterized by vesicular lesions on the hands, feet, and mouth. Unlike chickenpox, impetigo does not cause systemic symptoms, although it may cause pustules or vesicles. Chickenpox lesions manifest in many phases of development, whereas smallpox lesions are more consistent in their stages.

 In this case report, acyclovir was effective because it was recommended for adolescents, especially those aged 12 and above. Since calamine lotion reduces severe itching, stops skin infections, and helps dry off leaking skin, it is frequently used to treat chickenpox. Zinc oxide in calamine lotion, was serves as an antiseptic which, help cleanse the skin and get rid of bacteria [2]. 

 Acyclovir 800 mg taken orally five times a day for 10 days was used to treat the chickenpox infection because it prevents the varicella zoster virus from replicating. Acyclovir can sometimes eradicate the varicella zoster virus, but it can also resistant in some strains of the virus. After 7 to 10 days of acyclovir treatment, persistent lesions were considered to be resistant VZV. Its latency is established in human sensory neurons, specifically in the cranial root ganglia and dorsal root ganglia, where it remains dormant. Acyclovir typically isn't able to eradicate VZV from a latent state with neurons [4].

 Follow-up

 Healthcare providers monitor signs to make sure that he recovered without any issues related to complications. He recommended taking the medication as directed by a doctor and refraining from scratching lesions in order to prevent bacterial infections. The blisters and rash he had experienced completely healed and had no lasting complications. Usually, ten days pass before his rash goes away. He did not acquire dormant shingles, which is latent in neurons. He recovered without any issues like skin infections, encephalitis, pneumonia, or neurological problems. After a 10-day follow-up, he was sent home.

 References

 Presti CL, Curti C, Montana M, et al. Chickenpox: an update. Médecine et Maladies Infectieuses. 2019 Feb 1;49(1):1-8.

 Sharma P, Hasan MR, Mehto NK et al.: 92 years of zinc oxide: has been studied by the scientific community since the 1930s—an overview. Sensors International. 2022; 3:100182.

 Kennedy PG, Grinfeld E, Gow JW. Latent varicella-zoster virus is located predominantly in neurons in human trigeminal ganglia. Proc Natl Acad Sci U S A. 1998 Apr 14;95(8):4658-62.

# Reviewer report: Reviewer response for version 1

**DOI:** 10.5256/f1000research.137210.r158332

**Published:** 2023-01-05

**Authors:** Anna Majewska

**Affiliations:** 1 Department of Medical Microbiology, Medical University of Warsaw, Warszawa, Poland

The case description lacked some information:

1. How many days did the rash/blisters last?
2. Were any tests performed (differential diagnosis)?
3. Is the author sure about the diagnosis of VZV? Long-term skin lesions are unusual for immunocompetent individuals
4. Has the patient been in contact with a person infected with VZV?
5. Whether treatment with acyclovir was effective?

Moreover,

1. I think that laboratory test confirming the diagnosis should be performed (PCR, culture, antibody titre test)?
2. Currently, the use of powder liquids as preparations for smallpox lesions is abandoned, because bacteria can multiply under their application. Perhaps the rash was a result of bacterial superinfection.
3. The discussion section is incorrectly written. 4. "...acyclovir inhibits the the duplication of the varicella zoster virus, and has the ability to eradicate varicella zoster ...,," --replication instead of duplication would be a better word and aciclovir has no ability to eradicate VZV, latency is established in the neurons.

Are enough details provided of any physical examination and diagnostic tests, treatment given and outcomes?

No

Is the case presented with sufficient detail to be useful for other practitioners?

No

Is sufficient discussion included of the importance of the findings and their relevance to future understanding of disease processes, diagnosis or treatment?

No

Is the background of the case’s history and progression described in sufficient detail?

Partly

Reviewer Expertise:

Virology, infectious diseases, herpesviruses, antiviral drugs

I confirm that I have read this submission and believe that I have an appropriate level of expertise to state that I do not consider it to be of an acceptable scientific standard, for reasons outlined above.

# Response

**Published:** 2024-11-17

**Authors:** Gudisa Tola

**Affiliations:** negelle health science college, Ethiopia

Reviewer 1

 The need for a chickenpox modification method has become clearer since reports of a number of serious and sometimes fatal complications have been made public. These include primary varicella pneumonia in adults, visceral dissemination, neonatal chickenpox death, and others [1].

 While chicken pox spots develop over a few days, mpox spots emerge all at once. In general, mpox lesions are deeper and larger than chickenpox lesions. While chickenpox blisters are simply itchy, mpox blisters can cause agony. Spots of mpox will simultaneously blister and crust over. With crusty sores, blisters, and chickenpox patches all at once. There are two distinct viruses that produce chickenpox and mpox. Chickenpox in this case report was confirmed by laboratory diagnosis [2, 3].

 What this study adds

 This study provides comprehensive knowledge and insights about less common transmission of the varicella zoster virus (VZV), which causes chickenpox, via contaminated surfaces. In this case report, VZV was spread by coming into contact with surfaces contaminated with the virus via blister fluid from an infected person. Surface-contaminated route of transmission, when compared with direct contact with lesions and airborne distribution, was thought to be a less significant mechanism of transmission. This instance is occurring in a surprising age bracket, particularly in healthy adults who are not immunocompromised and have never had chickenpox.

 How this study might be useful for other practitioners

 A more thorough examination of patient risk variables will help clinicians identify patients, particularly young adults, who may be more susceptible to problems. How they provided supportive care, such as controlling pruritus, managing fever, and taking care of the skin to avoid secondary infections. It included comprehensive instructions on how to avoid and treat complications like encephalitis, pneumonia, and bacterial infections, which could help medical professionals provide preventative care.

 Physical examinations

 Throughout the assessment, his blood pressure was 121/81 mmhg, his axillary temperature was 38.4 degrees Celsius, his peripheral pulse rate was 78 beats per minute, and his respiration rate was 19 cycles per minute. His blood tests revealed that his hemoglobin was 15.1 mg/dl, his white blood cell count was 10540 cells/mm3, his red blood cell count was 4.7 million/mm, and his neutrophil count was 59%. Blood urea nitrogen was 44 mg/dl, creatinine was 98 μmol/L, alkaline phosphatase was 215 U/L, gamma glutamyl transferase was 19 U/L, and alanine transferase was 9 IU/L. His liver and kidney function tests showed no appreciable acute alterations.

 The x-ray of his chest showed no signs of community-acquired pneumonia. Examinations of his heart, lungs, and abdomen revealed no noteworthy results. Results from polymerase chain reaction tests for VZV or skin lesions (a vesicle or scab material) were positive. He tested positive for VZV with both his skin swab and serology (IgG) antibodies. Serology (IgM) antibodies were positive, indicating a high level of recent VZV infection.

 Differential diagnosis

 In this case report, the differential diagnosis of chickenpox includes smallpox, drug eruptions, hand, foot, and mouth disease, herpes simplex virus, and disseminated herpes zoster. Grouped vesicles, particularly on the lips or vaginal area, are caused by the herpes simplex virus. As opposed to being ubiquitous like chickenpox, it typically affects specific locations. Although drug eruptions frequently feature mucosal lesions and systemic symptoms different from chickenpox, they can also result in a widespread vesicular or bullous rash. There was no sign of angioedema, skin necrosis, or erosions of the mucosal membranes in the patient. Before the rash appeared, the patient had not started taking any medicines.

 Disseminated herpes zoster, also known as shingles, is a reactivation of the varicella zoster virus that does not initial infection in chickenpox and occurs in older or immunocompromised patients. Hand, foot, and mouth disease (HFMD), as opposed to a broad body rash, is characterized by vesicular lesions on the hands, feet, and mouth. Unlike chickenpox, impetigo does not cause systemic symptoms, although it may cause pustules or vesicles. Chickenpox lesions manifest in many phases of development, whereas smallpox lesions are more consistent in their stages.

 Follow-up

 Healthcare providers monitor signs to make sure that he recovered without any issues related to complications. He recommended taking the medication as directed by a doctor and refraining from scratching lesions in order to prevent bacterial infections. The blisters and rash he had experienced completely healed and had no lasting complications. Usually, ten days pass before his rash goes away. He did not acquire dormant shingles, which is latent in neurons. He recovered without any issues like skin infections, encephalitis, pneumonia, or neurological problems. After a 10-day follow-up, he was sent home.

 References

 Ross AH: Modification of chicken pox in family contacts by administration of gamma globulin.N Engl J Med. 1962; 267: 369-76 

 Rasizadeh R, Shamekh A, Shiri Aghbash P, et al.: Comparison of human monkeypox, chickenpox, and smallpox: A comprehensive review of pathology and dermatological manifestations. Current Medical Research and Opinion. 2023;39(5):751-60.

 Class MM, McCoy K, Hafeez F, et al. Case Letter. Cutis. 2024 Oct;114(4): E29-31.
